# The Impact of Diagnostic Code and Inclusion Period on Clinical Outcomes in ATTR Cardiomyopathy

**DOI:** 10.1016/j.jacadv.2025.101918

**Published:** 2025-07-23

**Authors:** Pei-Lun Lee, Kuan-Yu Chi, Yu-Shiuan Lin, Dimitrios Varrias, Snehal R. Patel

**Affiliations:** a Jacobi Medical Center, Albert Einstein College of Medicine, Bronx, New York, USA; b Yale School of Medicine, New Haven, Connecticut, USA; c Center for Evidence-Based Medicine, Taipei Medical University Hospital, Taipei, Taiwan; d Montefiore Medical Center, Albert Einstein College of Medicine, Bronx, New York, USA

We read with great interest the study by Jaiswal et al^1^, which found that the use of sodium-glucose cotransporter 2 inhibitors (SGLT2i) was associated with prognostic benefits in transthyretin amyloid cardiomyopathy (ATTR-CM). Given the uncertain efficacy of conventional guideline-directed medical therapy for heart failure (HF), we commend the authors for their important contribution to advancing the field. While these findings are compelling, several methodological aspects of the study merit further discussions before drawing conclusions around SGLT2i use in ATTR-CM.

First, the reliance of the International Classification of Diseases-10th Revision-Clinical Modification code of E85.4 (organ-limited amyloidosis) to define ATTR-CM may introduce significant misclassification. This code encompasses amyloidosis involving various organs beyond the heart, including the kidneys, liver, and spleen. More importantly, this is not specific to transthyretin amyloidosis, potentially allowing the inclusion of patients with light-chain amyloidosis, which has distinct pathophysiology and treatment implications.^2^ The inclusion of non-ATTR-CM patients could have diluted or confounded the observed treatment effect of SGLT2i, limiting the generalization of the findings to the ATTR-CM population. Second, the study inclusion period (2000-2023) may have led to temporal bias. SGLT2i were approved in 2013, meaning the non-SGLT2i group included patients from 2000 to 2023, while all SGLT2i users were necessarily included after 2013. This imbalance is clinical relevance in ATTR-CM as the diagnostic timeline from HF symptom onset to ATTR-CM diagnosis, a well-recognized prognostic factor in ATTR-CM, has significantly shortened over this period, from a median of 3 years at early 2000 to 1 year after 2012.^3^ Additionally, HF management guidelines have evolved considerably over this time frame. The discrepancy in inclusion periods between the 2 groups may have contributed to selection bias, affecting the precision of the reported treatment effect of SGLT2i in ATTR-CM.

In summary, while this observational study offers intriguing insights, the results should be interpreted with caution due to the potential biases introduced by the study design.
